# NDRG3 overexpression is associated with a poor prognosis in patients with hepatocellular carcinoma

**DOI:** 10.1042/BSR20180907

**Published:** 2018-12-07

**Authors:** Ji-sheng Jing, Hongbo Li, Shun-cai Wang, Jiu-ming Ma, La-qing Yu, Hua Zhou

**Affiliations:** 1 Department of Infectious Diseases, Jurong People’s Hospital Affliated to Jiangsu University, Zhenjiang, Jiangsu, P.R. China; 2 Department of Hepatology, Infectious diseases Hospital Affliated to Soochow University, Suzhou, Jiangsu, P.R. China

**Keywords:** HCC, IHC, NDRG3, PCR, prognosis

## Abstract

N-myc downstream-regulated gene 3 (NDRG3), an important member of the NDRG family, is involved in cell proliferation, differentiation, and other biological processes. The present study analyzed NDRG3 expression in hepatocellular carcinoma (HCC) and explored the relationship between expression of NDRG3 in HCC patients and their clinicopathological characteristics. We performed quantitative real-time reverse-transcription polymerase chain reaction (qRT-PCR) analysis and immunohistochemistry (IHC) analyses on HCC tissues to elucidate NDRG3 expression characteristics in HCC patients. Kaplan–Meier survival curve and Cox regression analyses were used to evaluate the prognoses of 102 patients with HCC. The results revealed that compared with non-tumor tissues, HCC tissues showed significantly higher NDRG3 expression. In addition, our analyses showed that NDRG3 expression was statistically associated with tumor size (*P*=0.048) and pathological grade (*P*=0.001). Survival analysis and Kaplan–Meier curves revealed that NDRG3 expression is an independent prognostic indicator for disease-free survival (*P*=0.002) and overall survival (*P*=0.005) in HCC patients. The data indicate that NDRG3 expression may be considered as a oncogenic biomarker and a novel predictor for HCC prognosis.

## Introduction

Hepatocellular carcinoma (HCC) is one of the most common malignancies worldwide. Among malignant tumors, HCC ranks fifth in prevalence and second in mortality rate globally [1]. In 2015, over 20,000 mortality cases of HCC occurred in the United States [2]. In China, new cases and mortality cases of HCC comprise more than half of the global total (>400,000 cases) [3]. Qidong county, in Eastern China’s Jiangsu Province, has the highest HCC prevalence worldwide [4]. HCC occurrence and development are multi-factorial, multi-stage, and continuous processes that are pertinent to factors such as hepatitis B, hepatitis C viral infection, alcohol damage, and non-alcoholic fatty liver [5,6]. In the last two decades, HCC therapeutics such as chemotherapy, microwave ablation, surgical resection, radiofrequency, and liver transplantation [7] have progressed, yet the high metastasis and recurrence rates of HCC illustrate that its overall prognosis remains unsatisfactory [8]. Considering this, and the complexity of prognosis-related factors, prognostic predictors must be identified to improve the clinical treatment of HCC.

N-myc downstream-regulated gene 3 (NDRG3) is a major member of the NDRG family and plays important roles in cell proliferation, differentiation, and other biological functions [9,10]. NDRG3 is highly expressed in the testicles, ovaries, prostate, spinal cord, and primitive thymus, and its transcription product is the most highly expressed in the brain, followed by the heart and kidneys [11]. NDRG3 exists in the outer layers of the seminiferous epithelium, indicating that it may play a role in spermatogenesis [12]. In recent years, the complex roles of NDRG3 in carcinogenesis have attracted attention. NDRG3 promotes cell growth in prostate cancer tissues. Excessive NDRG3 expression in cells can up-regulate angiogenesis factors, such as CXCL1, CXCL3, and CXCL5, and their main role is to augment blood vessel growth to promote tumor growth [13,14]. NDRG3 is overexpressed in hepatitis B virus (HBV)-related hepatocellular carcinoma, and as such, it is a potential therapeutic target for HCC [15]. NDRG3 overexpression is also thought to be correlated with non-small cell lung cancer (NSCLC), prostate cancer, and laryngeal squamous cell carcinoma [16–18]. These findings suggest that NDRG3 has tumor-promoting effects; however, a recent report indicated that NDRG3 down-regulation might be involved in breast cancer occurrence and progression to an advanced stage [19]. However, no study has investigated the prognostic value of NDRG3 in HCC, which thus requires further elucidation.

In the present study, we used quantitative real-time reverse-transcription polymerase chain reaction (qRT-PCR) to analyze NDRG3 expression in HCC specimens and their neighboring non-tumor tissues. In addition, we performed tissue microarrays (TMA) and immunohistochemistry (IHC) to examine NDRG3 expression in HCC tissues. Finally, we also evaluated the relationship between NDRG3 expression and the clinicopathological features of HCC patients, specifically the relationship between NDRG3 expression and its prognostic characteristic.

## Materials and methods

### Patients and specimens

Twenty fresh-frozen HCC samples and the matching tumor-adjacent tissues samples were enrolled from the People’s Hospital of Jurong Affiliated with Jiangsu University, and subjected to qRT-PCR between January 2015 to May 2016. A total of 102 cases of HCC were enrolled to construct tissue microarrays (TMA) from the People’s Hospital of Jurong, Jiangsu, China, between February 2006 and October 2011. All 102 patients were diagnosed with Hepatocellular carcinoma and clinical data (including age, gender, tumor size, tumor encapsulation, tumor number, liver cirrhosis, Pathological grade, Hepatitis B virus infection, Vascular invasion, TNM stage, overall survival (OS), disease-free survival (DFS), and 5-year follow-up survival records) were obtained from the medical records of each patient. None of the patients had received adjuvant therapies before surgery. The TNM stages were defined according to 2010 AJCC staging system for HCC. The present study is retrospective and was approved by the Ethics Committee of Jurong People’s Hospital.

### Quantitative real-time reverse transcription PCR (qRT-PCR)

qRT-PCR was executed as described elsewhere [20]. The primers for NDRG3, designed by BioTNT Co., Ltd (Shanghai, China), were as follows: NDRG3,forward: 5′-CCA GGA CTT TGACTG TCA GGA-3′ and reverse: 5′-AGT GCTGGG TGA TCT CTT GC-3′, β-actin, forward: 5′-TTA ATC TTCGCC TTA ATA CTT-3′ and reverse: 5′-AGC CTT CAT ACA TCT CAA-3′. The relative expression of NDRG3 was indicated by −∆*C*_t_ value. All measurements were performed in triplicate.

### Tissue microarrays and immunohistochemistry (IHC)

One hundred and two specimens were selected randomly and TMAs were constructed from the representative two cores from each specimen. The tissue cores were cut into 4 µm sections and was then placed onto Superfrost charged glass microscope slides. IHC was performed as described in previous study [21,22], Specimens were incubated with anti-human NDRG3 rabbit monoclonal antibody (1:200, ab133715, Abcam, Cambridge, MA) in phosphate-buffered saline (PBS). Afer being washed with PBS, the slides were performed with anti-rabbit secondary IgG antibody. PBS was used instead of the primary anti-NDRG3antibody as a negative control. The IHC score ranged from 0 to 12, which was generated by number of staining cells proportion multiply staining cell intensity. The density of NDRG3 in staining cells was graded as follows: 0 (negative staining), 1 (yellow staining), 2 (light brown staining), and 3 (dark brown staining). Staining percentage of NDRG3 was categorized as follows: 0 (0%), 1 (1–25%), 2 (11–50%), 3 (51–75%), and 4 (76–100%). The IHC score ranged from 0 to 12, which was generated by number of stained cells multiply staining intensity. The cut-off point for a high NDRG3 expression score in terms was set using the median IHC score (4.5). Low and high expression of NDRG3 were defined as IHC score ≤4 and >4, respectively. Immunohistochemical staining was evaluated and scored by two independent pathologists, without knowledge of the clinicopathological and follow-up information of these patients.

### Statistical analysis

The SPSS software package, version 18 (SPSS Inc, Chicago, IL, U.S.A.) and STATA 14.0 (Stata Corporation, College Station, TX, U.S.A.) were used for statistical analyses. Data were analyzed by sample-paired *t*-test to compare the differential expression levels of NDRG3 mRNA between tumor and adjacent non-tumor liver tissues of HCC patients.

Expression of NDRG3 protein was estimated using the Wilcoxon signed rank nonparametric test because the data were not normally distributed. The associations between NDRG3 expression and clinicopathologic parameters were calculated by chi-square tests. Kaplan–Meier method was used to generate survival curves and the differences were analyzed using the log-rank test. Cox regression model was established for the multivariate survival analysis to determine prognostic factors that were signifcant on univariate analysis for either DFS and OS. Significance was established when *P*<0.05.

## Results

### Clinical characteristics of 102 HCC patients

In the present study, a tissue microarray (TMA) containing one hundred and two cases of HCC and non-cancerous samples was obtained from the Affiliated Jurong People’s Hospital of Jiangsu University (Zhenjiang, China) to conducted IHC analysis, including 18 females and 84 males with a median age of 52 years (27–84 years). Forty-nine subjects had tumor diameters >5 cm, while the remaining 53 had tumor diameters ≤5 cm. Examination of their disease history revealed that 86 subjects had HBV infection, and 90 had liver cirrhosis. Pathological staging analysis showed that 2 subjects were stage I, 61 were stage II, and 39 were stage III. Fifty-three subjects had tumor capsule. Sixty-six patients were tumor, node, metastasis (TNM) stage I, 33 were stage II, and 3 were stage III (Table 1).

**Table 1:** Relationship between NDRG3 overexpression and the clinicopathological characteristics of HCC patients

Groups	No.	NDRG3		χ^2^	*P* value
		+	%		
Total	102	50	49.0		
Gender					
Male	84	42	50.0	0.18	0.669
Female	18	8	44.4		
Age (years)					
≥60	26	13	50.0	0.01	0.908
<60	76	37	48.7		
Tumor size (cm)					
>5	49	29	59.2	3.90	0.048*
≤5	53	21	39.6		
Tumor encapsulation					
None	49	25	51.0	0.15	0.698
Complete	53	25	47.2		
Tumor number					
Multiple	16	9	56.3	0.40	0.529
Solitary	86	41	47.7		
Hepatitis B virus infection					
Yes	86	41	47.7	0.40	0.529
No	16	9	56.3		
Liver cirrhosis					
Yes	90	45	50.0	0.29	0.588
No	12	5	41.7		
Pathological grade					
Grade 1-2	63	23	36.5	10.32	0.001*
Grade 3	39	27	69.2		
Vascular invasion					
Present	31	20	64.5	5.35	0.069
Absent	66	29	43.9		
Insufficient data	5	1			
TNM stage					
Stage I	66	29	43.9	2.75	0.253
Stage II	33	20	64.5		
Stage III	3	1			

* *P*<0.05.

### Examination of NDRG3 mRNA in HCC tissues by qRT-PCR

**Figure 1:**
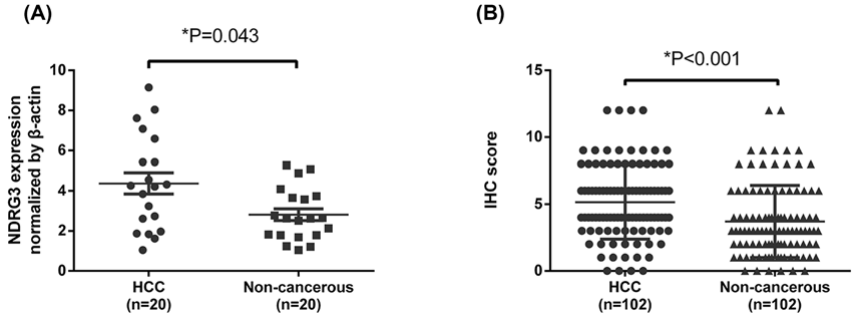
NDRG3 was up-regulated in hepatocellular carcinoma (HCC) (**A**) NDRG3 mRNA was markedly increased in tumor tissues than that in the paired adjacent nontumor tissues. *P=0.043, paired *t*-test. (**B**) The IHC score of NDRG3 was indicated by Dot distribution graph. Data are mean ± SD (Wilcoxon signed-rank test).

To examine the NDRG3 mRNA levels in HCC patients, qRT-PCR assays were performed using 20 pairs of samples of HCC tissues and matching adjacent tissue. As shown in Figure 1A, our results, which used β-actin as the internal control for normalization, revealed that HCC tissues had significantly higher NDRG3 expression (4.37 ± 0.527) than that of their neighboring non-tumor tissues (2.81 ± 0.292) (mean ± SEM values, *P*<0.05).

### Examination of NDRG3 expression in HCC tissues by IHC

**Figure 2:**
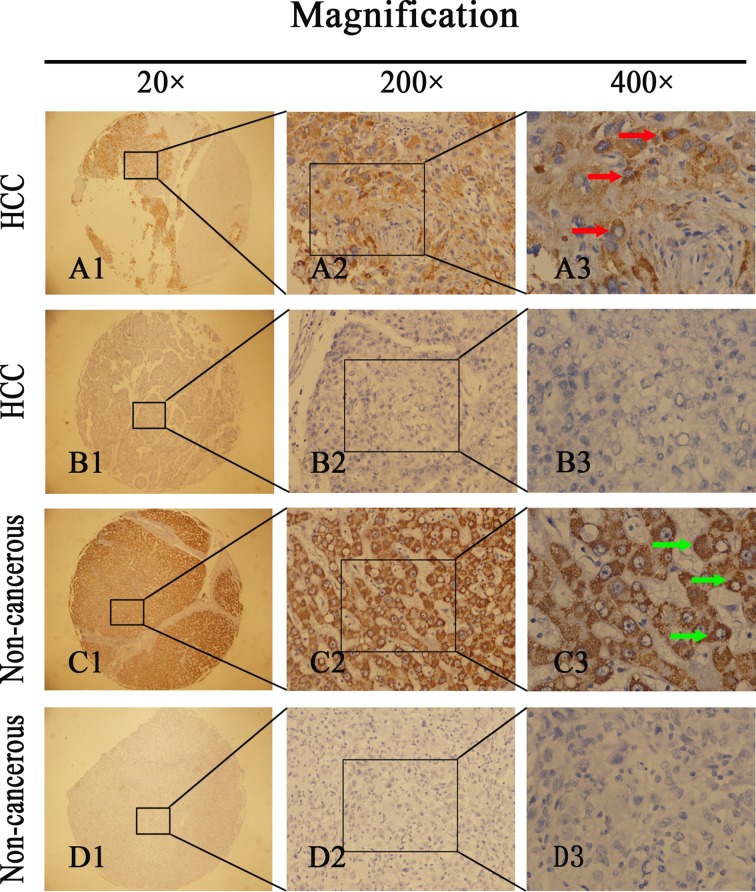
Immunohistochemistry (IHC) assays of NDRG3 expression in 102 paired HCC and adjacent non-tumorous tissues A1, A2, A3 represents high NDRG3 expression in HCC. Red arrow shows positive staining in the membrane and cytoplasm of cancer cells. B1, B2, B3 represents low NDRG3 expression in HCC. C1, C2, C3 and D1, D2, D3 represents high and low NDRG3 expression in adjacent non-tumorous tissues. Green arrow shows positive staining in the membrane and cytoplasm of non-tumorous cells. Original magnification: ×20 in A1–D1; ×200 in A2–D2; ×400 in A3–D3.

IHC was performed to determine NDRG3 expression in HCC tissues. Of the 102 HCC specimens, 50 manifested NDRG3 overexpression (49.0%); however, of the 102 non-tumor specimens, only 27 (26.5%) had positive NDRG3 expression. Compared with non-tumor tissues, HCC tissues showed significantly higher levels of NDRG3 protein (Figure 1B) (*P*<0.001). Positive staining for NDRG3 was primarily localized in the cytoplasm and cell membrane. Figure 2 shows a classical IHC staining NDRG3 pattern in HCC cells. Table 1 shows the relationship between NDRG3 protein levels and HCC clinicopathological features. Statistical analyses revealed that positive expression of NDRG3 was significantly correlated with tumor size (*P*=0.048) and pathological grade (*P*=0.001) but not with sex, age, tumor capsule, tumor number, hepatitis B surface antigen, liver cirrhosis, vascular invasion, or TNM staging.

### Survival analysis

**Figure 3:**
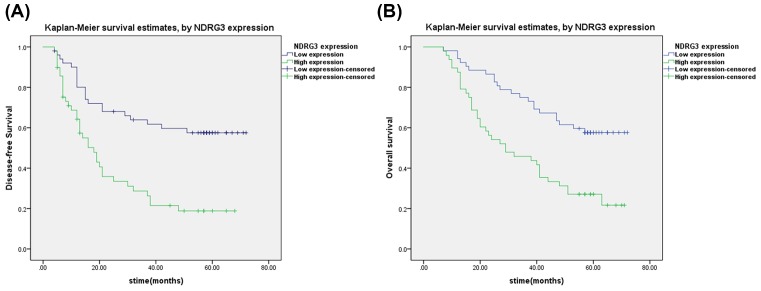
Survival analysis of hepatocellular carcinoma (HCC) patients by Kaplan-Meier method. High NDRG3 expression is associated with poor outcomes of patients with HCC. Kaplan–Meier analysis disclosed the significant differences in disease-free survival (*P*<0.001) (**A**) and overall survival (*P*<0.001) (**B**) between postoperative patients with high and low NDRG3 expression in HCC cohort Survival analysis of HCC patients by Kaplan–Meier method. The *P*-values were determined using the log-rank test.

A Cox regression univariate analysis revealed that some factors were associated with DFS of HCC patients, including NDRG3 expression, tumor size, vascular invasion, and TNM staging (Table 2). All the above items were also associated with OS in 102 HCC patients (Table 3). A multivariate analysis was performed, which revealed that NDRG3 (*P*=0.002) indicated a poor DFS, whereas NDRG3 expression (*P*=0.005) and tumor size (*P*=0.033) were two independent predictors of prognosis for OS (Tables 2 and 3). In addition, Kaplan–Meier curves revealed that HCC patients with high NDRG3 expression presented a significantly unfavorable DFS time (*P*<0.001) and OS time (*P*<0.001) (Figure 3A,B).

**Table 2:** Univariate and multivariate analyses of the predictors of disease-free survival in HCC patients

	Univariate analysis			Multivariate analysis		
	HR	*P* > |*z*|	95% CI	HR	*P* > |*z*|	95% CI
NDRG3 expression						
High versus Low	2.81	0.001*	1.626–4.846	2.62	0.002*	1.417–4.829
Gender						
Male versus Female	1.97	0.093	0.893–4.356			
Age (years)						
≥60 versus <60	0.92	0.763	0.513–1.632			
Tumour size (cm)						
>5 versus ≤5	2.05	0.007*	1.213–3.481	1.51	0.173	0.835–2.735
Tumor encapsulation						
None versus Complete	1.17	0.544	0.698–1.977			
Tumor number						
Multiple versus Solitary	1.52	0.216	0.784–2.930			
Hepatitis B virus infection						
Yes versus No	1.12	0.753	0.564–2.210			
Liver cirrhosis						
Yes versus No	1.02	0.94	0.462–2.246			
Pathological grade						
Grade 1 and 2 versus Grade 3	0.94	0.811	0.542–1.614			
Vascular invasion						
Present versus Absent	2.02	0.012*	1.165–3.517	1.21	0.870	0.127–11.432
TNM stage						
Stage I versus Stage II versus Stage III	0.47	0.002*	0.291–0.751	0.78	0.811	0.098–6.169

* *P*<0.05.

**Table 3:** Univariate and multivariate analyses of the predictors of overall survival in HCC patients

	Univariate analysis			Multivariate analysis		
	HR	*P* > |*z*|	95% CI	HR	*P* > |*z*|	95% CI
NDRG3 expression						
High versus Low	2.53	0.001*	1.486–4.323	2.37	0.005*	1.301–4.306
Gender						
Male versus Female	1.79	0.126	0.849–3.780			
Age (years)						
≥60 versus <60	1.05	0.86	0.591–1.875			
Tumour size (cm)						
>5 versus ≤5	2.25	0.003*	1.329–3.813	1.87	0.033*	1.053–3.331
Tumor encapsulation						
None versus Complete	1.15	0.592	0.688–1.928			
Tumor number						
Multiple versus Solitary	1.35	0.37	0.700–2.606			
Hepatitis B virus infection						
Yes versus No	1.23	0.557	0.620–2.427			
Liver cirrhosis						
Yes versus No	1.10	0.811	0.499–2.430			
Pathological grade						
Grade 1 and 2 versus Grade 3	0.89	0.659	0.515–1.522			
Vascular invasion						
Present versus Absent	1.85	0.029*	1.066–3.203	1.26	0.839	0.135–11.753
TNM stage						
Stage I versus Stage II versus Stage III	0.53	0.005*	0.344–0.831	0.59	0.616	0.075–4.648

* *P*<0.05.

## Discussion

N-myc downstream regulatory genes are a newly discovered gene family comprising NDRG1, 2, 3, and 4. By modulating target gene transcription, NDRG components influence cell proliferation, apoptosis, differentiation, and disease onset [23,24]. As such, they play crucial roles in tumorigenesis in humans [25,26]. Phylogenic analysis demonstrated that human NDRG1 and NDRG3 belong to one subfamily, whereas NDRG2 and NDRG4 belong to another [10]. Currently, different opinions exist on NDRG1 expression in tumors. One study showed that NDRG1 might become a tumor suppressor in colorectal cancer (CRC) by inhibiting tumor growth or inducing cell apoptosis. Specifically, NDRG1 can block MARCH-8-induced degradation of receptor 4, and CRC cells expressing NDRG1 are more sensitive to reagents that target death receptors such as tumor necrosis factor-related apoptosis-inducing ligands (TRAIL) [27]. NDRG1 overexpression in esophageal squamous cell carcinoma (ESCC) is also reported to be associated with short OS in these patients. NDRG1 overexpression in ESCC cells is reported to activate the Wnt signaling pathway to induce the epithelial–mesenchymal transition (EMT), thereby reducing E-cadherin expression but increasing Snail expression [28]. Tumor suppressor NDRG2 depends on repressing E3 ubiquitin ligase Skp2 activity to induce CRC cell differentiation [29]. Hypoexpression of NDRG2 may also activate the NF-κB signaling pathway to induce EMT, thereby significantly increasing the quantity and size of oral squamous cell carcinomas (OSCCs) as well as the likelihood of invasion into the neck lymph nodes [30]. Limited studies on NDRG3 and NDRG4 revealed that NDRG4 plays tumor suppressive roles in colorectal cancer and glioblastoma [31–33]. NDRG3 properties have been reported in several cancers. *In vitro* overexpression of exogenous NDRG3 in PC-3 cells is reported to led to increased clone numbers, migration capabilities and growth rates compared with parental or mock empty vector transfected PC-3 cells, and *in vivo* overexpression of NDRG3 elevated xenograft tumor growth in nude mice. These findings suggest that NDRG3 plays a key role in prostate cancer occurrence and development [13]. Fan et al. [15] reported that NDRG3 was up-regulated in HCC specimens and that inhibiting NDRG3 could reduce the malignant HCC cell phenotype. A recent study showed that NDRG3 up-regulation was detected in both NSCLC specimens and cell lines; thus, its expression may be a novel predictor of NSCLC [18]. NDRG3 studies mainly focus on determining its biological mechanisms. Lactate-induced responses through the NDRG3–Raf–ERK axis are reported to be conducive for maintaining tumor progression under long-term hypoxia [34–36]. Evidence has revealed that NDRG3 is a new and useful biomarker for human cancer; however, despite reports that NDRG3 is up-regulated in HCC specimens, its clinicopathological relevance and relationship to HCC prognosis remain unclear. More studies are needed to examine NDRG3 as a potential therapeutic target of HCC.

Our qRT-PCR results indicated that HCC specimens had higher mRNA levels of NDRG3 than did normal non-tumor tissues. Likewise, TMA and IHC analyses also showed that NDRG3 protein levels in HCC tissues was tremendously elevated than that of non-cancerous. These findings were consistent with previous studies [15–18] and collectively indicated that NDRG3 expression is up-regulated in malignant tumors. In addition, positive NDRG3 expression in HCC specimens is positively correlated with some clinicopathological parameters (e.g., tumor size and pathological grade). Du et al. [37] proposed that miR-31 upregulation may reduce HCC occurrence by suppressing NDRG3 expression, which is consistent with our previous results. These results further reveal that NDRG3 may play an key role in HCC occurrence.

Univariate and multivariate analyses revealed that NDRG3 expression was associated with DFS of HCC patients and can potentially be used as independent predictors of DFS, whereas NDRG3 expression and tumor size were identified as independent prognostic factors that affect OS. In addition, Kaplan–Meier analysis also demonstrated that patients with positive NDRG3 expression had shorter life spans than their counterparts with negative NDRG3 expression. The results were in accordance with the findings of previous studies, which found that NDRG3 overexpression in tumors promoted tumor development and was associated with a poor prognosis [16–18].

In contrast, some studies have indicated that NDRG3 expression was decreased in some tumor types, such as breast cancer, and thus appeared to function as tumor suppressors [19]. This discrepancy might be due to differences in tumor origins and the potential

functional differences of NDRG3 in different tumor types, as in the case of NDRGs [38–40].

The present study has some limitations. For example, we did not detect NDRG3 expression in tumor cells from lymph nodes, which may be pivotal for the involvement of NDRG3 in HCC growth and metastasis. In addition, the present study was based on TMA specimens, which were limited and lacked information on TNM Stage IV and HCV infection or the history of alcohol consumption of the HCC patients; thus, these results may be biased. We will continue to improve our experimental design. In summary, this study examined differences in NDRG3 expression in HCC specimens and, for the first time, the association of NDRG3 expression and clinical attributes of HCC patients, especially the prognostic function of NDRG3 was addressed. NDRG3 is considered a new prognostic marker for HCC patients and may yield new therapeutics for HCC patients.

## Abbreviations

95% CI: 95% confidence interval
AJCC: American Journal of Critical Care
CRC: colorectal cancer
CXCL: C-X-C motif chemokine ligand
DFS: disease-free survival
EMT: epithelial–mesenchymal transition
ESCC: esophageal squamous cell carcinoma
HBV: hepatitis B virus
HCC: hepatocellular carcinoma
HR: hazard ratio
IHC: immunohistochemistry
mRNA: messenger RNA
NDRG: N-myc downstream-regulated gene
NSCLC: non-small cell lung cancer
OS: overall survival
OSCC: oral squamous cell carcinoma
PBS: phosphate-buffered saline
qRT-PCR: quantitative real-time reverse-transcription polymerase chain reaction
TMA: tissue microarrays
TNM: tumor node metastasis
